# Volatile Fatty Acids (VFA) Production and Recovery from Chicken Manure Using a High-Solid Anaerobic Membrane Bioreactor (AnMBR)

**DOI:** 10.3390/membranes12111133

**Published:** 2022-11-11

**Authors:** Dong Min Yin, Clarisse Uwineza, Tugba Sapmaz, Amir Mahboubi, Heleen De Wever, Wei Qiao, Mohammad J. Taherzadeh

**Affiliations:** 1 Swedish Centre for Resource Recovery, University of Borås, 501 90 Borås, Sweden; 2 Biomass Engineering Center, College of Engineering, China Agricultural University, Beijing 100083, China; 3 Changzhou Key Laboratory of Biomass Green, Safe & High Value Utilization Technology, Institute of Urban and Rural Mining, Changzhou University, Changzhou 213164, China; 4 Flemish Institute for Technological Research, VITO NV, Boeretang 200, B-2400 Mol, Belgium

**Keywords:** immersed membrane bioreactor, volatile fatty acids, chicken manure, acidogenic fermentation

## Abstract

Acidogenic fermentation of chicken manure (CM) for production and recovery of volatile fatty acids (VFA) is an interesting biological waste-to-value approach compared to benchmark organic waste management strategies. Considering the wide range of high value applications of VFA, a semi-continuous immersed anaerobic membrane bioreactor (AnMBR) was applied to boost VFA productivity and yield, while reducing downstream processing stages assisting the recovery of VFA. In this regard, the effect of parameters such as pH and organic loading rates (OLR) on the overall bioconversion and filtration performance was investigated. Thermal-shocked CM was applied both as inoculum and substrate. A very high VFA yield (0.90 g-VFA/g-VS) was obtained in the treatment with no pH control (~8.2) at an OLR of 2 g-VS/(L·d), presenting 24% higher yield compared to that of the controlled pH. Batch assays further demonstrated the enhanced hydrolysis and acidogenesis activities at weak alkaline conditions. A long-term (78 days) fermentation and filtration was successfully performed, where stable membrane filtration performance was experienced for about 50 days under high-solid (suspended solid of 37–45 g/L) and high flux (20 L/(m^2^·h)) conditions. Results suggest that AnMBR of CM is a feasible and promising process for VFA production and recovery.

## 1. Introduction

Statistics show that in 2019, the global stock of laying hens was 3.85 billion, and the number of broilers was about 50 billion [1], leading to a huge amount of chicken manure (CM) production of around 267 million tons (around 40 kg of manure per laying hen per year and 3 kg of manure from each broiler) [2]. CM is considered as a promising bioresource for energy recovery through anaerobic digestion (AD) technology. AD has been established as a promising organic waste handling method; biogas, the main product of AD formed of mainly methane and carbon dioxide, is an energy carrier which can be produced through biological conversion of organic matter. However, the high content of nitrogen in CM causes serious ammonia inhibition of methane production while reducing the organic conversion rates [3,4]. In an AD process using chicken manure as substrate, when increasing the substrate and ammonium loading (up to 8.5 g/L), the methane yield declined by 94%, while volatile fatty acids (VFA) accumulation increased more than 60 times [3,5]. Therefore, the AD of high-solid CM comes along with challenges such as low methane conversion efficiency, and if diluted to a low feeding concentration result in a large amount of digestate difficult to deal with [6]. Although ammonia inhibition affects methanogenic activity in a negative way, acid production through anaerobic fermentation might benefit from the inherent characteristics of chicken manure with higher organic loading rates and ammonium concentration.

Volatile fatty acids, including acetic acid, propionic acid, butyric acid, iso-butyric acid, valeric acid, iso-valeric acid and caproic acid, as the most important intermediate products of anaerobic digestion, have enjoyed increasing interest in a wide range of industrial applications [7]. For instance, acetic acid and propionic acid are used in food and beverages industries, butyric acid in textiles and bioenergy industries, iso-butyric acid in cosmetics, valeric and iso-valeric acid in perfumes, and caproic acid in the production of rubber and grease [7,8]. In addition, studies in recent years have shown that mixed VFA can be used as a carbon source to cultivate fungi for animal feed [7]. Mixtures of acetic acid and butyric acid have been used in the synthesis of biodiesel and acetic, propionic and butyric acid have been used in the synthesis of biopolymers or to cultivate algae [9]. VFA are conventionally provided through petrochemical routes; however, this entails issues associated with the overexploitation and application of fossil energy and chemical sources. Provision of VFA through green and sustainable alternative approaches such as AD of organic waste can act as future chemical and material platforms. Simultaneously, in theory, the conversional process in AD from organic wastes to VFA instead of methane can be promoted through not only enhancing the hydrolysis/acidogenesis activity by control the pH [10,11] but also through inhibiting VFA consumption, by inhibiting the activities of methanogens [7]. Many research studies have reported the acidogenic fermentation (AD with the inhibition of the methane formation step) of different substrates, such as food waste [12], citrus waste [13], straw [14], municipal sludge [15] and cow manure [16,17]. However, these processes have not been extended to continuous operations or scaled up to assess their suitability for engineering applications [18,19,20], or they have not been efficient in the recovery of VFA-rich streams from the complex effluent [19,21]. At present, the commonly used technology for VFA collection from the digestate is the energy and cost intensive process of centrifugation.

In recent years, membrane separation technology has received widespread attention as an advantageous technique for continuous separation and recovery of valuable compounds such as fuels, pharmaceuticals and VFA from bioreactors [22,23,24]. Using a membrane bioreactor (MBR) not only assists with the recovery of a VFA solution with low turbidity [25], but also reduces the effects of product accumulation promoting the progression of bioconversion. Moreover, an MBR used in an AD system can well prevent the wash-out of microorganisms, ensuring a continuous and stable operation [13,26]. Previous research has reported maximum obtainable VFA yields through continuously operated MBRs of about 0.14–0.80 g-VFA/g-VS_fed_ dealing with different substrates, for example, swine manure [27], Napier grass [28] and food waste [29,30]. However, the difference in the characteristics of the substrate used for acidogenic fermentation can greatly affect the complex filtration and fermentation function of an MBR, further affecting production yield [31,32]. To the authors’ knowledge, there are few studies on acidogenic fermentation and VFA separation from CM using anaerobic MBRs (AnMBR). Therefore, there is a great lack of information on appropriate adjustment of AnMBR parameters to maximize VFA output, as regards increasing the organic loading rate (OLR) and conversion efficiency and reducing economic losses caused by filtration-associated problems, etc.

In the current research, a high-solid AnMBR was set up to investigate acidogenic fermentation from CM to VFA and in situ recovery of VFA-bearing effluent. The effect of factors such as pH (control at 6.0 and uncontrolled) and OLR on the overall process performance and outcome were thoroughly studied. The hydrolysis and acidification kinetics were evaluated through batch assays. In addition, mass balancing was used to reveal the influence of pH on microbial activity. The membrane filtration performance and membrane fouling were monitored and characterized through the changes of permeate flux, medium viscosity, solid content and transmembrane pressure (TMP).

## 2. Materials and Methods

### 2.1. Substrate, Inoculum and Thermal Shock Pretreatment

The raw CM was collected from an egg-laying farm Sjömarkens Hönsgård AB (Borås, Sweden), with total solid (TS) and volatile solids (VS) contents of around 327 and 220 g/L, respectively. To physically inhibit methane formation, optimal thermal shock pretreatment assays were carried out [20]. The raw CM was firstly diluted with tap water to VS of around 100 g/L and mixed with a heavy-duty blender (Waring^®^ CB15, Front Royal, VA, USA) at medium speed for around 15 s. The CM was then thermally treated at different temperatures (80 °C, 90 °C, 100 °C, 110 °C and 120 °C) for 30, 60, 90, 120, 150 and 180 min, separately (Figure S1 in Supplementary Materials). Based on the relatively high ratio of soluble chemical oxygen demand (SCOD) to total chemical oxygen demand (TCOD), the condition of heating at 80 °C for 90 min was chosen to pretreat the CM prior to feeding. The obtained slurry was then sieved (aperture of around 0.1 mm), analyzed and stored at −20 °C until use. Some characteristics of the sieved thermally shocked CM were as follows: pH of about 7.8 and TS, VS, total suspended solid (TSS) and dissolved solids (DS) of around 63.9, 45.1, 37.8 and 25.4 g/L, respectively. The VFA content of the slurry was 4.5 g/L (including acetic acid 3.8 g/L) and NH_4_^+^-N content was 4550 mg/L, referring to previous results [20]. CM was heat shocked (80 °C, 15 min), sieved, and diluted to VS of around 10 g/L to be used as inoculum in the current research. The characteristics of the substrate and inoculum are presented in Table 1.

### 2.2. Experimental Set Up and Operational Parameters

**Figure 1:**
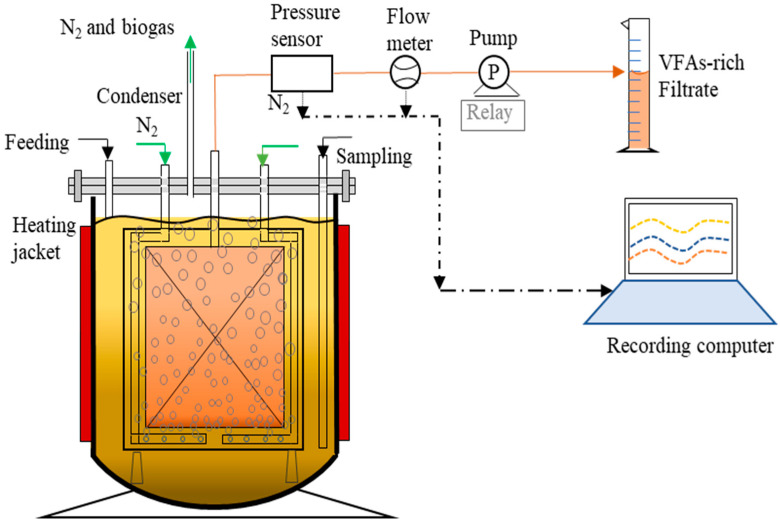
Schematic diagram of the applied Anaerobic Membrane bioreactor (AnMBR).

As shown in Figure 1, two laboratory-scale semi-continuous AnMBRs were operated with active working volumes of 3.5 L (total 4 L) operated at 37 °C and HRT of 10 d. The reactors operated in parallel. One benefited from controlled pH at 6.0 (AnMBR1) (pH control solutions used: 2 mol/L HCl and/or NaOH) while. in the other, pH was left uncontrolled (AnMBR2). In both reactors, the membrane panels applied were constructed from hydrophilic polyether sulfone (PES)/PVP membrane layers casted on either side of a spacer fabric by VITO NV (Mol, Belgium). The membranes had an average pore size of 0.3 μm and each panel had an active membrane surface of 205.8 cm^2^. The maximal permeability was tested as 3000–4000 L/(m^2^·h·bar) with deionized clean water. Six built-in pores with a diameter of 0.5 mm were arranged at the bottom of the membrane panel to scour the membrane surface. Nitrogen sparging at a flow rate of 5 L/min was applied to scour the membrane surface during filtration and 3 L/min for better medium mixing during the fermentation cycle. The permeate flow and TMP were recorded using ultrasonic flowmeters (Atrato 710-V11-D, Titan Enterprises Ltd., Sherborne, UK) and pressure transmitters (Endress+Hauzer, Reinach, Switzerland), using a MEFiAS monitoring and control program developed by VITO NV. 

Initially, 3.5 L of inoculum (heat-shocked CM, VS of 100 g/L) was filled into AnMBR1 and AnMBR2, separately, followed by pH adjustment to 6 for AnMBR1. During the startup phase (days 0–7), reactors were operated in batch mode until the VFA in the inoculum were consumed, reaching a stable level (2.3 g/L). After the startup phase, AnMBRs were shifted to semi-continuous mode with initial feeding of 20 g-VS/L from day 8 to 53 (OLR of 2 g-VS/(L·d)), followed by a period of OLR 4 g-VS/(L·d) from day 54 to 78 (feeding VS of 40 g-VS/L). For AnMBR2, pH was recorded once per day prior to feeding. 15 mL of well mixed effluent was sampled every day for the analysis of medium characteristics, such as viscosity, TS, VS, TSS, VSS, TCOD, and SCOD. Permeate containing VFA was filtered out (335 mL) on daily basis followed by feeding (350 mL) pretreated CM. The filtration process consisted of a 6 min filtration cycle (flow rate of 20 mL/min) followed by a 30 s backwash cycle (flow rate of 60 mL/min) for physical membrane cleaning. The concentration of VFA and ammonium nitrogen (NH_4_^+^-N) in the permeate was analyzed every day. To remediate filtration related issues, SS draining was applied at different time intervals.

To evaluate VFA stripping from the reactor due to gas sparging, evaporation tests were carried out. Solutions consisting of acetic acid, propionic acid and butyric acid (ratio of 7:1.5:1.5) with different initial pH (6.0 and 8.0) and initial total concentrations (10 and 20 g/L) at different nitrogen gas-flow rates (3 and 5 L/min) were used for evaporation experiments (Figure S2 and Table S1 in Supplementary Materials). Sampling was performed every 30 min from two reactors for a total of 5 h. Ratios between the evaporated VFA and aeration time can be assumed as the coefficient of VFA during filtration and fermentation cycle (Table 2). VFA evaporation correction has been considered in all VFA results presented.

### 2.3. Hydrolysis, Acidogenesis, Acetogenesis, Methanogenesis and Hydrogenesis Kinetics under Different pH

Batch assays were conducted in 120-mL serum bottles at 37 °C to explore the kinetics of hydrolysis, acidification and methanogenesis in AnMBR1 and AnMBR2. Around 120 mL of sludge was taken from AnMBR1 and AnMBR2, respectively, during three consecutive days, i.e., 40–42th for OLR 2 g-VS/(L·d), analyzing the VSS of the well-mixed sludge for calculation. 10 mL of sludge and 90 mL of basic anaerobic medium [4] were added into the serum bottles. Kinetics were assessed after adding 10 g-COD/L soluble starch (hydrolysis activity), 10 g-COD/L glucose (acidogenesis activity) and 3 g/L sodium acetate (methanogenesis and hydrogenesis activities) to the sludge and basic anaerobic medium. Batch reactors incubated with 10 mL of inoculum sludge and 90 mL of basic anaerobic medium solution without starch/glucose/sodium acetate were used as controls. The 2 mol/L HCl or NaOH were used to set the pH of the reactors in the range of the continuous reactors (6.0 or 8.2). The batch experiments were triplicated for both AnMBR1 and AnMBR2. Batch reactors were incubated at 37 °C in water bath shakers at 100 rpm. Analysis of biogas yield and composition (H_2_, CH_4_ and CO_2_) was conducted on the 2nd, 4th, 6th, 8th and 10th days, while a 2 mL aliquot of the fully mixed digestate was withdrawn from day 0 to 10 every two days for SCOD or VFA analysis.

Hydrolysis and acidogenesis activities were calculated based on the substrate consumption rate according to Equations (1) and (2) [33]:

(1) $$Hydrolysis=-\frac{1}{\rho \left(VSS\right)}\cdot \frac{d\rho \left(SCOD+COD_{CH_{4}}+COD_{H_{2}}\right)}{dt}\;(g\text{-}\mathrm{COD}/(g\text{-}\mathrm{VSS}\cdot d))$$

(2) $$Acidogenesis=-\frac{1}{\rho \left(VSS\right)}\cdot \frac{d\rho \left(COD_{VFA}+COD_{CH_{4}}+COD_{H_{2}}\right)}{dt}(g\text{-}\mathrm{COD}/(g\text{-}\mathrm{VSS}\cdot d))$$

Methanogenesis and hydrogenesis kinetics were calculated based on the methane and hydrogen production rate according to Equations (3) and (4):

(3) $$Methanogenesis=\frac{1}{\left(VSS\right)}\cdot \frac{d\rho \left(COD_{CH_{4}}\right)}{dt}\;(g\text{-}\mathrm{COD}/(g\text{-}\mathrm{VSS}\cdot d))$$

(4) $$Hydrogenesis=\frac{1}{\left(VSS\right)}\cdot \frac{d\rho \left(COD_{H_{2}}\right)}{dt}(g\text{-}\mathrm{COD}/(g\text{-}\mathrm{VSS}\cdot d))$$

where the VSS was the volatile suspended solids, the COD*_VFA_* was calculated by the oxygen demand of individual VFA, and the COD*_CH_4__* and COD*_H_2__* were calculated based on the conversion factors of 350 mL CH_4_/g-COD and 1400 mL H_2_/g-COD under standard condition, ρ shows the concentration of each parameter, and t being the fermentation time, in days.

### 2.4. Analytical Methods

TS and VS were measured by drying and burning samples of specific volume in an oven and muffle furnace at 105 °C and 550 °C, respectively, following the standard methods of the American Public Health Association [34]. The concentrations of TCOD, SCOD and NH_4_^+^-N were determined with test kits (Nanocolor, MACHEREY-NAGEL GmbH & Co., KG, Düren, Germany) and Nanocolor 500D Photometer (MACHEREY-NAGEL GmbH & Co., KG, Düren, Germany) [16,20]. Free ammonia nitrogen (NH_3_-N) was calculated based on NH_4_^+^-N and pH [4]. VFA concentrations and gas composition (H_2_, CH_4_ and CO_2_) were analyzed using Gas Chromatography (GC) (Clarus 550; Perkin-Elmer, Norwalk, CT, USA) as described previously [16]. The volumetric gas values were converted to standard conditions at a pressure of 1.01 bar and a temperature of 273.2 K. Statistical significance of the obtained results was calculated using one-way analysis of variance (ANOVA, *p* < 0.05). In order to record medium viscosity, 10 mL reactor medium from AnMBR1 and AnMBR2 was withdrawn and used for measurement in a Vibro viscometer (SV-10, A&D Co., Ltd., Tokyo, Japan) at 37 °C. Total nitrogen and protein content of raw CM, feed and effluent during the stable stage were measured with a Kjeldahl nitrogen analyzer (KD210 Opsis Co., Ltd., Skytteskogsvgen, Sweden) and calculated based on Equation (5):

(5) $$TKN\;\left(\%\right)=\frac{C\times \left(V_{1}-V_{2}\right)\times 14}{1000\times m_{0}}\times 100$$

where *C* was the concentration of HCl, 0.01 mol/L; *V*_1_ was the volume of HCl consumed in titrating manure samples, mL; *V*_2_ was the volume of HCl consumed in titrating blank sample, mL; *m*_0_ was the initial mass of samples before drying, g.

## 3. Results and Discussions

A semi-continuous mesophilic AnMBR was applied to investigate VFA production and in situ recovery performance using heat shocked CM as both substrate and inoculum. In this regard, the effects of pH and OLR on VFA yield and productivity as well as on long-term membrane filtration performance were studied. In addition, the kinetic activities of hydrolysis, acidogenesis, acetogenesis and methanogenesis and mass balance based on changes in COD levels were determined.

### 3.1. Effects of pH and OLR on VFA Fermentation

**Figure 2:**
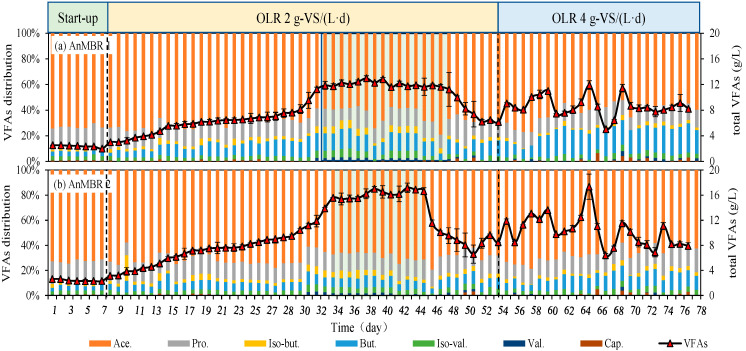
Changes in the VFA production and distribution in the (**a**) AnMBR1 and (**b**) AnMBR2.

As presented in Figure 2, after a 7-d startup period with nitrogen sparging (3 L/min), the VFA concentration in heat-shocked pretreated CM decreased gradually from 3.1 g/L to 2.0 and 2.3 g/L in AnMBR1 and AnMBR2, respectively. With the start of the semi-continuous operation and daily feeding of the reactors under OLR 2 g-VS/(L·d), VFA productions showed a linear increase, reaching and stabilizing at 16.2 g/L, by day 35 in AnMBR2. In addition, the evaporated VFA were recorded to be at around 1.8 g/L for AnMBR2 at fermentation pH of around 8.0 and initial total VFA of 20 g/L (Table 2). Therefore, it can be extrapolated that the original VFA production from CM was about 18.0 g/L in AnMBR2 at OLR of 2 g-VS/(L·d). However, relatively lower VFA were present in AnMBR1 at controlled pH 6.0 (14.5 g/L), by day 32 at OLR of 2 g-VS/(L·d), (2.5 g/L of evaporated VFA have been compensated according to Table 2). The average VFA yields for AnMBR1 and AnMBR2 at OLR 2 g-VS/(L·d) were around 0.73 and 0.90 g-VFA/g-VS_in_, respectively. These yields, no matter with or without pH control, were much higher than those of many other organic wastes, such as cow manure (0.39–0.15 g-VFA/g-VS at OLR from 0.8–4.7 gVS/(L·d)) [16], food waste (0.54 g-VFA/g-VS at OLR of 2 gVS/(L·d) or 0.52 g-VFA/g-VS at OLR of 6 gVS/(L·d)) [29,35]. However, stability in VFA production was only maintained for 16 d (day 32–47) for AnMBR1 and 11 d (day 35–45) for AnMBR2. VFA concentration dropped rapidly to 6.1 and 8.5 g/L in AnMBR1 and AnMBR2 after a stable phase while the reactors still operated at OLR 2 g-VS/(L·d). Continuous decline in VFA concentrations may suggest that organic loading was deficient [3,16]. On the other hand, as the fermentation progressed, the activity of methanogens may have gradually recovered, resulting in more conversion of VFA to biogas [5]. However, a solid conclusion in this regard requires further investigation, as in this study, the AnMBRs were continuously purged with nitrogen gas, diluting the gas produced due to microbial activity such as methane below the detection limit of the analytical equipment. In order to boost VFA accumulation in the system, the OLR was doubled to 4 g-VS/(L·d) (feeding VS 40 g/L) on day 54. Following the increase in loading, acidogenic fermentation experienced a slight increase. However, soon the average concentration of VFA in both AnMBR1 and AnMBR2 reduced to between 12.9–13.3 g/L, ending lower than that of the stable phase at OLR 2 g-VS/(L·d) (Table 3). The change of OLR gave an initial boost to VFA production due to higher loading and initial increases in the ammonium content; however, as previously reported [16], this was followed by a lower conversion rate and lower yield of metabolite on the volatile solid loaded. In agreement with the observations in this study, it has been reported that in a submerged AnMBRs an increase in TSS due to poor substrate degradability or high loading hinders proper mixing and mass transfer in addition to filtration related issues [6]. However, when organic loading and in response VFA production is high, the in-situ recovery of VFA-rich filtrate through the membrane is essential in order to prevent the inhibition of microbial activity by the presence of high acid concentration [6,16].

Regardless of the stage of fermentation, loading rate or pH, acetic, propionic and butyric acids were the most abundant VFA (Figure 2), of which, acetic acid formed the highest VFA fraction (65–70%), followed by propionic acid (about 10%) and butyric acid (5–10%). This result was consistent with the results acquired in batch assays using CM [20].

pH is one of the key factors controlling metabolic pathways and ultimately determining the distribution of VFA [36]. Butyric acid and propionic acid are reported to be dominant at low pH, while acetic acid becomes the main metabolic product at high pH [37]. It has also been claimed that the increase in pH from 4.0 to 7.0 reduces the concentration of butyric acid, while production of acetic acid is enhanced [15]. However, in the current research, the changes in the pH had a more significant effect on the total amount of VFA produced than their distribution. The proportion of propionic acid was similar to the results of previous fermentations of VFA produced from co-fermentation of cow manure [15,38], chicken manure and silage corn stover [17]. In summary, high VFA yields of up to 0.90 g-VFA/g-VS from pretreated CM present the potential of AnMBR fed with CM for the establishment of a VFA platform.

### 3.2. Effects of Ammonia Nitrogen on VFA Yield from CM

**Figure 3:**
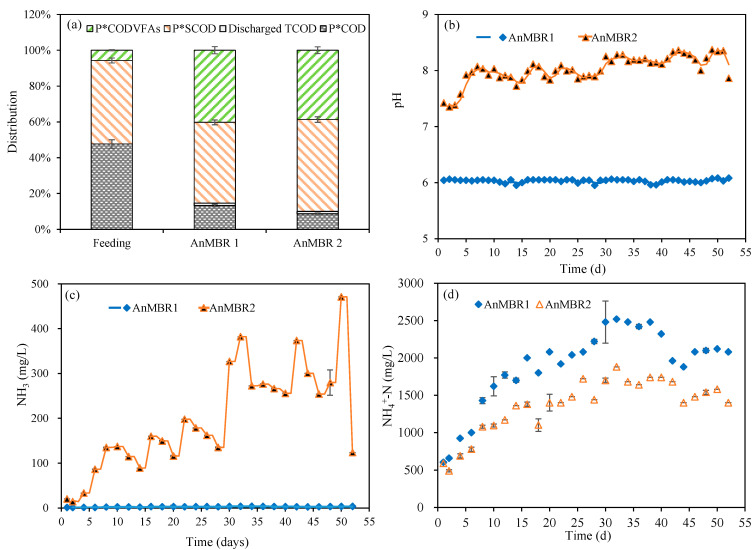
(**a**) Mass balance based on COD during the VFA production and changes of (**b**) pH, (**c**) NH_4_^+^-N, and (**d**) NH_3_ at OLR 2 g-VS/(L·d).

Mass balances for as-received CM, pretreated CM and effluent withdrawn from AnMBR1 and AnMBR2 were performed based on COD content during the stable stage at OLR 2 g-VS/(L·d) (Figure 3a). As expected, P*COD (undegraded organic matters, i.e., the part of TCOD deducted from SCOD) is reduced as CM is pretreated. The decrease in P*COD, from 85.9% in as-received CM to 52.6% in the pretreated slurry, presents a hydrolysis rate of 33.3% of the organic matter after pretreatment at 80 °C for 90 min. This result was in accordance with previous reports on thermal pretreatment of cow slurry at 94–100 °C for 30 min and its ability to double the concentration of SCOD compared with untreated cow slurry [15]. This hydrolysis due to pretreatment increased the organic matter in the liquid phase of CM, reducing the load of organics in the solid fraction. The hydrolysis and acidification rates were further improved in the AnMBR feed and effluent when compared on COD balance basis. The remaining unconverted organic matter in AnMBR1 and AnMBR2 was 13.2% and 4.2%, respectively. In addition, due to daily samplings, the fraction of discarded TCOD was 2.2%. Consistent with the higher VFA concentrations in AnMBR2 compared to AnMBR1, the proportion of COD*_VFA_* in AnMBR2 reached up to 46.8% (around 10.2% higher than AnMBR1). S*COD represents the proportion of SCOD excluding the contribution of VFA. The SCOD levels in AnMBR1 and AnMBR2 were 84.6% and 93.7%, respectively (Figure 3a). The significant increase in the concentration of SCOD and VFA proves the promising hydrolysis and acidification in AnMBRs.

During the anaerobic digestion of CM, VFA, especially acetic acid and propionic acid, can be formed during the degradation of proteins and amino acids [15,39]. The total Kjeldahl nitrogen content of as-received CM was 26.3% (Table 1), which was much higher than that of other manure such as cow manure (2.9%) [16]. It can be assumed that the composition of VFA was affected by the digestion of nitrogen-containing compounds in CM. The released NH_4_^+^-N in the AnMBR is mainly due to the hydrolysis of proteins and amino acids. At OLR 2 g-VS/(L·d), the average concentrations of ammonia nitrogen in AnMBR1 and AnMBR2 were 2262 and 1645 mg/L, respectively (Table 3). As the pH in AnMBR2 showed a gradual increasing trend with the extension of the reactor operating time, from 7.9 at the initial feeding stage to 8.3 (Figure 3b), the corresponding free ammonia nitrogen gradually increased, from initial 86 to about 471 mg/L (Figure 3c,d). As for AnMBR1, with a constant pH control throughout the fermentation, the content of free ammonia was kept close to zero. The increase in NH_3_-N in AnMBR2 is the main reason why the NH_4_^+^-N in AnMBR2 was lower than AnMBR1 (Figure 3d). It has been reported in other literature that, although, ammonia nitrogen can be used as an essential nutrient for microbial growth, it may inhibit microbial activity (mainly methanogens) at high concentrations [4,40]. It has been reported that as the concentration of total ammonia nitrogen (free ammonia and ammonium) reaches levels higher than 1.7 g/L, it inhibits methanogenesis [3,4]. This can be taken as a natural method for methane production inhibition and VFA accumulation. On the other hand, during the process of acidogenic fermentation, the accumulation of VFA could possibly be neutralized by NH_4_^+^-N, thereby stabilizing the pH during digestion [41]. It can be seen that the yield of VFA in AnMBR1 under pH controlled at 6.0 was significantly lower than that of AnMBR2 (pH of about 8.2), which could be due to the inhibition of the activity of proteolytic bacteria under acidic conditions [31]. The protein content of the bioreactors was about 10 and 2 g/kg in AnMBR1 and AnMBR2 during the stable stage at OLR 2 g-VS/(L·d), corresponding to protein degradation rates of 37.5% and 87.5% in AnMBR1 and AnMBR2, respectively. The relative low protein conversion rate in AnMBR1 may have contributed to the lower VFA yields in comparison to AnMBR2.

### 3.3. Kinetic Activities at Different pH

**Figure 4:**
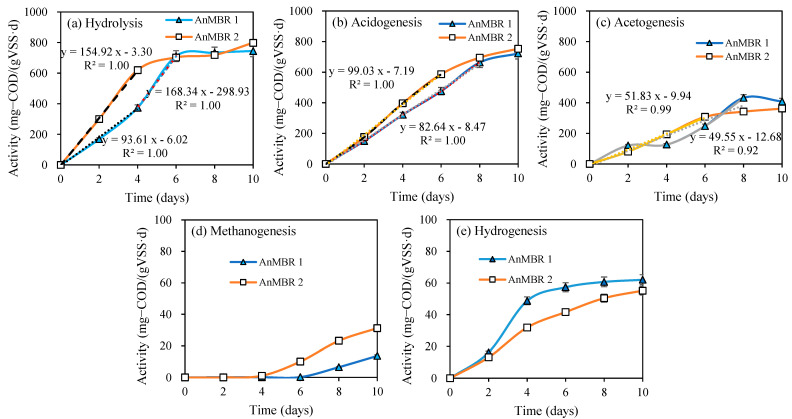
(**a**) Hydrolysis, (**b**) acidogenesis, (**c**) acetogenesis, (**d**) methanogens and (**e**) hydrogenase activities of AnMBR1 and AnMBR2.

In order to determine the maximum four-steps’ kinetic activities of the sludge, batch assays were carried out applying the sludge from the stable stage at OLR 2 g-VS/(L·d) of AnMBR1 and AnMBR2. A significant increase in rates was observed in both AnMBR1 and AnMBR2 with the extension of fermentation time (Figure 4). The hydrolysis activities of the sludge from AnMBR1 and AnMBR2 both showed a linear increase during the first four days of AD at a rate of 93.6 and 154.9 mg-COD/(g-VSS·d), respectively. The hydrolysis activities obtained for AnMBR1 and AnMBR2 were comparable after 6 d of AD (Figure 4a). The hydrolysis activity slowly increased to the maximum of 798 mg-COD/(g-VSS·d) on day 10, with the conversion rate of 80.9% for sludge from both reactors (Figure 4a). For AnMBR1, the hydrolysis activity increased sharply during the 4th to 6th day, at a rate of 168.3 to 711 mg-COD/(g-VSS·d), slightly higher than that of AnMBR2, indicating that the sludge in the AnMBR1 reactor was inhibited to a certain extent during the first few days. The hydrolysis process was usually reported as a rate-limiting step during anaerobic digestion for high-solid organic matter such as the CM (TS of 30–45%) used in this work [12,33,42]. Greatly improved hydrolysis activity can effectively promote the conversion efficiency of solid organic matter into soluble and easy-to-use smaller organic matter, positively improving the yields of final products, e.g., VFA. Similar trends were found for acidogenic activity in the two reactors. At OLR 2 g-VS/(L·d), the hydrolysis and acidogenic activities were very close for both reactors. At the same time, only slightly higher acidification activity was found for sludge from AnMBR2, reaching 753 mg-COD/(g-VSS·d) by day 8 (Figure 4b). Additionally, the rates of acidogenesis for both were close to that of hydrolysis, presenting the balance between SCOD release and consumption, and VFA production [4]. In contrast with a previous report by Yin et al. (2021), pH 6.0 was conducive to the accumulation of VFA, with the type of inoculated sludge being a possible reason. Research on acidogenic fermentation from cow manure has demonstrated that the type of inoculated sludge will significantly affect the production and yield of VFA [16]. Thus, it was inferred that acidic conditions could be advantageous for acid-producing fermentation with granular sludge, while alkaline pH (uncontrolled) might be more conducive to the substrate conversion when manure itself was used as inoculum. The hydrolysis activities of CM under mesophilic and thermophilic conditions (HRT 20 days) were previously reported to be around 230–260 mg-COD/(g-VSS·d) [4], which presents a potential of only about 32–36% of the results achieved in the current research.

The methanogenic and hydrogenesis activities of the two reactors at OLR 2 g-VS/(L·d) are shown in Figure 4c,d. In comparison with the hydrolytic and acidogenic activities, much lower methanogenic and hydrogenesis activities were recorded. It might be that the thermal shock pretreatment enhanced the hydrolysis process along with inhibiting the methanogens activity [42]. In addition, the presence of ammonium and VFA at the reported concentrations has positively assisted the inhibition of methanogenic activity of the sludge in AnMBR1 and AnMBR2. There were no methanogenic activities until day 4, while they marginally increased to 14 and 31 mg-COD/(g-VSS·d) for AnMBR1 and AnMBR2 by day 10, respectively. Compared to the alkaline condition provided in AnMBR2, the activity of methanogens was inhibited by about 50% at pH 6.0 (AnMBR1). This result was consistent with the result in methane producing processes [43]. Consequently, the hydrogenesis activities under the condition of pH 6.0 was higher by 12–53% compared with that under weak alkaline conditions (Table 4).

### 3.4. Membrane Filtration Performance

**Figure 5:**
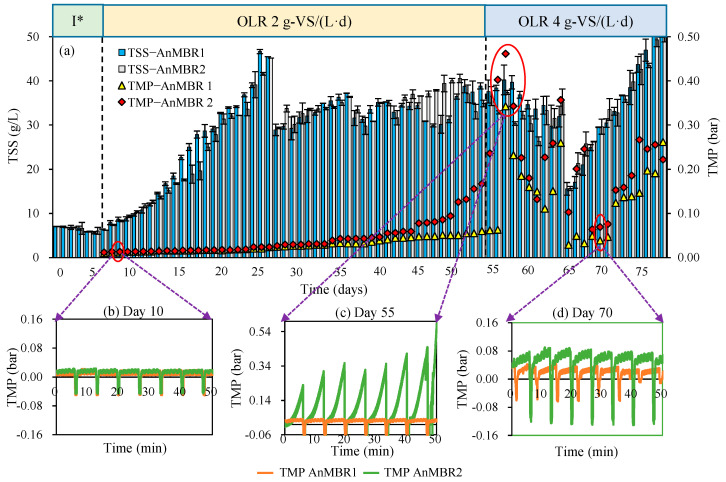
(**a**) Changes in the TMP and TSS during fermentation and filtration in the AnMBRs and the TMP at (**b**) day 10, (**c**) day 55, and (**d**) day 70. I* represents the start-up stage.

In the current research, the application of an immersed AnMBR ensured the production of VFA and in situ recovery of the VFA-bearing effluent during the acidogenic fermentation of CM. The semi-continuous AnMBR reactors operated for a total of 78 days (7 days startup included). With a feeding load of 20 g-VS/L, the TSS content of the reactor media increased from initially around 6 g/L to 46.7 and 41.6 g/L until day 28 at rates of 1.92 and 1.74 g TSS/(L·d), respectively, in AnMBR1 and AnMBR2 (Figure 5a). In order to maintain stable operation of the reactor, sludge drainage was used from day 28 to the end to maintain the TSS concentration at about 35 g/L in AnMBR1 and AnMBR2. Immersed membrane reactors are commonly applied in wastewater treatment with mixed suspended liquor (MLSS) content between 8 and 15 g/L [44]. The filtration operation at TSS up to 35–40 g-TSS/L in this study presents a high-solid AnMBR [40]. In addition, the initial membrane flux was set at 20 L/(m^2^·h), which is considered rather high compared to similar practices (<10 L/(m^2^·h)) using organic waste for VFA production [7,16,33].

**Figure 6:**
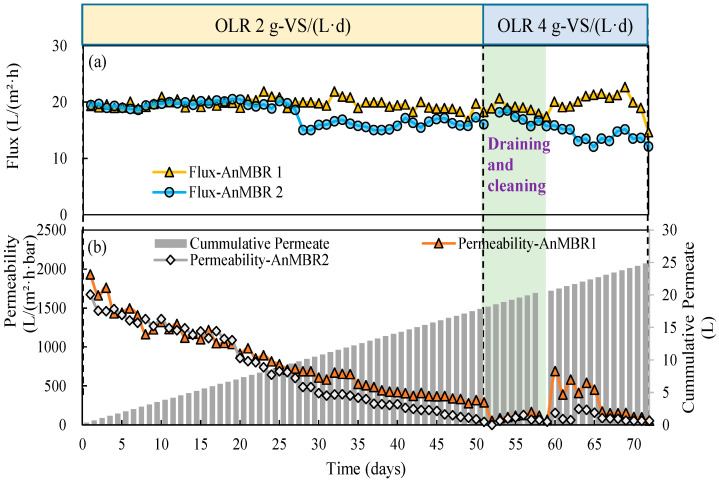
(**a**) Average flux and (**b**) permeability profiles in the AnMBRs during the filtration at OLRs 2 and 4 g-VS/(L·d).

In the current research, filtration in AnMBR1 and AnMBR2 was conducted smoothly and stably for 55 days under high-solid and high flux conditions (Figure 5a). As the filtration was not performed at either constant flux or TMP, changes in the membrane surface conditions and the extent of membrane fouling could easily be reflected in the changes of TMP and flux during operation. The TMP smoothly increased at rates of 0.93 and 1.57 mbar/d for AnMBR1 and AnMBR2, and reached 0.051 and 0.094 bar on day 50, respectively (Figure 5a,b). However, through 50 days of filtration, the permeability had dramatically decreased from initially 1930 to lower than 300 L/(m^2^·h·bar) due to the gradual increase in TMP in AnMBR1. This drop was even more pronounced for AnMBR2 as presented in Figure 6b. The gradual decline in permeability indicated that serious membrane fouling has occurred in the flat sheet membrane by gradual deposition of foulants on membrane surface forming a cake layer [45,46]. In this regard, physical fouling preventative remedies (backwashing, gas sparging and solid draining) were no longer effective. However, as destructive fouling detection practices were not possible in this case, further investigation in this regard is required to study fouling dynamics. Considering that CM is a complex substrate consisting of polysaccharides, suspended particles, colloids, proteins, microbial flocs and inorganic substances [47,48,49], the increase in TMP may be directly related to the continuous build up in TSS (Table 3). Such an outcome has been reported previously by Jomnonkhaow and his colleagues [16] using a similar AnMBR setup for acidogenic fermentation of cow manure. The ineffectiveness of physical membrane cleaning methods in this regard can be attributed to the formation of a rather compact cake layer or the development of biofilms and the deposition of biopolymers (such as extracellular polymers (EPS)) that are not easily separable by gas sparging or backwashing [50]. However, the sudden increase in TMP (Figure 5c) after day 45 could be a combined synergistic effect of a collection of medium-term characteristics and cake layer collapse and compaction [51,52,53].

## 4. Conclusions

This study comprehensively explored the process of acidogenic fermentation from chicken manure and in situ recovery of VFA from the reactor media in a high-solid AnMBR. Thermally shocked CM was proved to have great potential to be used as both inoculum and substrate for sustainable VFA production. High VFA yields of up to 0.90 g-VFA/g-VS at OLR 2 g-VS/(L·d) in a fermentation system with uncontrolled pH could be achieved. Although gradual membrane fouling could not be fully remediated, the immersed AnMBR was demonstrated to be capable of VFA production and recovery from CM under high flux and high solid load. The results clearly indicate the potential application of the AnMBR for direct VFA production and recovery from CM. However, further research in this area is to be carried out on the effects of higher organic loading rates on VFA generation and composition, and the exploration of potential applications for such mixed VFA effluents.

## Figures and Tables

**Table 1:** Characteristics of raw and pretreated chicken manure as inoculum and feeding.

	Units	Raw CM	Inoculum CM (80 °C, 15 min)	Sieved Thermally Shocked CM (80 °C, 90 min)
pH	-	-	7.1	7.8
TS	g/L	327 ± 2	14.6 ± 0.7	63.9 ± 4.9
VS	g/L	220 ± 1	10.1 ± 0.7	45.1 ± 0.2
VS/TS	%TS	67 ± 1	68.3 ± 2.4	70.5 ± 4.7
TSS	g/L	282 ± 5.0	7.1 ± 0.1	37.8 ± 0.9
VSS	g/L	183 ± 2	3.9 ± 0.1	25.4 ± 0.8
DS	g/L	45 ± 13.0	3.1 ± 0.7	26.1 ± 5.8
VS_D_	g/L	37 ± 1.0	3.1 ± 0.7	19.7 ± 0.6
TCOD	g/L	287 ± 4.0	12.8 ± 0.4	95.5 ± 0.7
SCOD	g/L	36 ± 2.0	5.5 ± 0.1	44.5 ± 0.7
Ace	g/L	3.2 ± 0.2	1.9 ± 0.3	3.8 ± 0.3
Pro.	g/L	0.3 ± 0.2	0.4 ± 0.2	0.5 ± 0.2
But.	g/L	0.2 ± 0.0	0.1 ± 0.0	0.2 ± 0.0
total VFA	g/L	3.8 ± 0.2	3.1 ± 0.1	4.7 ± 0.1
NH_4_^+^-N	mg/L	ND	495 ± 7	4550 ± 71
Bicarbonate alkalinity	mg-Ca(HCO_3_)_2_/L	ND	1063 ± 18	11,875 ± 355
Total alkalinity	mg-CaCO_3_/L	ND	1438 ± 35	12,500 ± 575
TKN	%	26.6 ± 0.3	ND	2.5 ± 0.1

Notes: CM: chicken manure, TS: total solids, VS: volatile solids, TSS: total suspended solids, VSS: volatile suspended solids, DS: minus of TS and SS, VSD: minus of VS and VSS, VFA: volatile organic acids, TCOD: total chemical oxygen demands, SCOD: suspended chemical oxygen demands, Ace.: acetic acid, Pro.: propionic acid, But.: butyric acid, TKN: total Kjeldahl nitrogen. SD: standard deviation (n means testing frequency), NA: not available, ND: not detected.

**Table 2:** Results of Volatile fatty acids (VFA) evaporation test under different conditions of pH, N_2_ sparging rates and initial VFA concentration.

	VFA (g/L)	Evaporation Coefficient (g/(L·h))				*t*_1_ (h)	*t*_2_ (h)	Volatile Concentration (g/(L·d))
		*k*_1_	*R* ^2^	*k*_2_	*R* ^2^			
AnMBR1 (pH 6.0)	10	−0.10	0.98	−0.13	0.99	22.5	1.5	2.5
	20	−0.14	0.99	−0.16	0.98	22.5	1.5	3.4
AnMBR2 (pH~8)	10	−0.07	0.89	−0.06	0.95	22.5	1.5	1.7
	20	−0.07	0.97	−0.12	0.96	21.5	2.5	1.8

Notes: The total volatile fatty acids (VFA) consisted of acetate: propionate: butyrate = 7:1.5:1.5; *k*_1_: the evaporation coefficient at time *t*_1_ (h) for fermentation cycling at gas flow rate of 3 L/min; *k*_2_: the evaporation coefficient time at time *t*_2_ (h) for filtration cycling at gas flow rate of 5 L/min.

**Table 3:** Changes in the medium characteristics in AnMBR1 and AnMBR2 at OLRs 2 and 4 g-VS/(L·d).

	Units	OLR 2 g-VS/(L·d)		OLR 4 g-VS/(L·d)	
		AnMBR1 (pH 6.0)	AnMBR2 (pH Uncontrolled)	AnMBR1 (pH 6.0)	AnMBR2 (pH Uncontrolled)
pH	\	6.0 ± 0.1	8.2 ± 0.2	6.0 ± 0.0	8.2 ± 0.3
TS	g/L	52.6 ± 6.9	43.1 ± 5.7	53.3 ± 16.2	46.5 ± 10.1
VS	g/L	28.4 ± 2.6	29.6 ± 2.6	33.7 ± 9.1	32.2 ± 7.1
VS/TS	%TS	54.7 ± 6.6	69.0 ± 2.9	63.9 ± 3.2	69.5 ± 4.9
TSS	g/L	33.0 ± 2.3	34.0 ± 2.5	34.8 ± 10.3	33.5 ± 8.5
VSS	g/L	25.1 ± 2.8	24.8 ± 1.4	26.2 ± 6.0	25.0 ± 5.0
TCOD	g/L	22.0 ± 5.6	21.7 ± 4.7	62.1 ± 6.1	68.7 ± 6.5
SCOD	g/L	13.4 ± 1.4	17.0 ± 1.5	21.6 ± 4.7	21.7 ± 4.7
TOC	g/L	6.0 ± 1.4	4.8 ± 0.6	6.0 ± 1.4	4.8 ± 0.6
Ace.	g/L	8.5 ± 0.7	11.9 ± 0.8	5.8 ± 1.3	7.0 ± 2.8
Pro.	g/L	2.3 ± 0.3	3.1 ± 0.2	1.8 ± 0.2	2.0 ± 0.4
But.	g/L	2.2 ± 0.2	1.4 ± 0.2	2.4 ± 0.3	1.3 ± 0.3
VFA *	g/L	14.5 ± 1.2	18.0 ± 0.7	13.3 ± 1.3	12.9 ± 0.7
NH_4_^+^-N	mg/L	2262 ± 236	1645 ± 146	2042 ± 518	1381 ± 307
NH_3_	mg/L	3.2 ± 0.4	269 ± 85	3.0 ± 0.9	245 ± 152
Viscosity	mPa·s	9.13 ± 1.04	3.03 ± 0.64	5.73 ± 3.90	3.50 ± 1.89
TKN	%	1.6 ± 0.1	0.3 ± 0.0	1.7 ± 0.1	0.2 ± 0.0

Notes: The data in table are the average values of stable periods (days 34–45 at OLR 2 g-VS/(L·d) and days 66–78 at OLR 4 g-VS/(L·d)). * The total VFA values shown were compensated for evaporation.

**Table 4:** Kinetic conversion efficiencies of AnMBR1 and AnMBR2 at OLR 2 g-VS/(L·d) (%).

Time (d)		2nd	4th	6th	8th	10th
AnMBR1	Hydrolysis (%)	16.8 ± 0.1	37.2 ± 0.2	65.0 ± 1.8	72.8 ± 1.7	73.9 ± 1.6
	Acidogenesis (%)	14.9 ± 0.1	31.9 ± 0.8	47.2 ± 0.8	65.9 ± 2.2	71.7 ± 2.2
	Methanogenesis (%)	0.0 ± 0.0	0.0 ± 0.0	0.0 ± 0.0	0.6 ± 0.0	1.4 ± 0.0
	Hydrogenesis (%)	1.3 ± 0.0	3.2 ± 0.1	4.1 ± 0.1	5.0 ± 0.0	5.5 ± 0.1
AnMBR2	Hydrolysis (%)	30.4 ± 0.3	62.8 ± 0.4	73.0 ± 2.5	77.0 ± 2.6	80.9 ± 2.3
	Acidogenesis (%)	17.9 ± 0.2	40.2 ± 0.3	59.5 ± 1.2	70.4 ± 2.1	76.4 ± 1.5
	Methanogenesis (%)	0.0 ± 0.0	0.1 ± 0.1	1.0 ± 0.1	2.4 ± 0.1	3.2 ± 0.1
	Hydrogenesis (%)	1.6 ± 0.0	4.9 ± 0.1	5.8 ± 0.2	6.2 ± 0.2	6.3 ± 0.1

## Data Availability

Data available on request from the corresponding author.

## Supplementary Materials

The following supporting information can be downloaded at: https://www.mdpi.com/article/10.3390/membranes12111133/s1, Figure S1: Hydrolysis efficiencies of chicken manure with thermal shock pretreatment under different temperature and time; Figure S2: VFA evaporation liner fit for different conditions of pH, N_2_ flow rates and initial VFA concentration; Table S1: VFA evaporation test during different conditions of pH. N_2_ flow rates and initial VFA concentrations for this work can be found in the e-version of this paper online.
