# Supplementary material for: Volatile Fatty Acids (VFA) Production and Recovery from Chicken Manure Using a High-Solid Anaerobic Membrane Bioreactor (AnMBR)
Source: Membranes (Basel). 2022 Nov 11;12(11):1133. doi: 10.3390/membranes12111133 (PMC9693206; doi:10.3390/membranes12111133)
Supplement: Supplementary file 1 [file membranes-12-01133-s001.zip › membranes-1974306-supplementary.pdf]

membranes

MDPI

*Supplementary Materials*

# Volatile Fatty Acids (VFA) Production and Recovery from Chicken Manure Using a High-Solid Anaerobic Membrane Bioreactor (AnMBR)

**Dong Min Yin [1,2,3], Clarisse Uwineza [1], Tugba Sapmaz [1], Amir Mahboubi [1], Heleen De Wever [4], Wei Qiao [2] and Mohammad J. Taherzadeh [1,*]**

1 Swedish Centre for Resource Recovery, University of Borås, 501 90 Borås, Sweden
2 Biomass Engineering Center, College of Engineering, China Agricultural University, Beijing 100083, China
3 Institute of Urban and Rural Mining, Changzhou Key Laboratory of Biomass Green, Safe & High Value Utilization Technology, Changzhou University, Changzhou 213164, China
4 Flemish Institute for Technological Research, VITO NV, Boeretang 200, B-2400 Mol, Belgium
* Correspondence: mohammad.taherzadeh@hb.se; Tel.: +46-033-435-5908

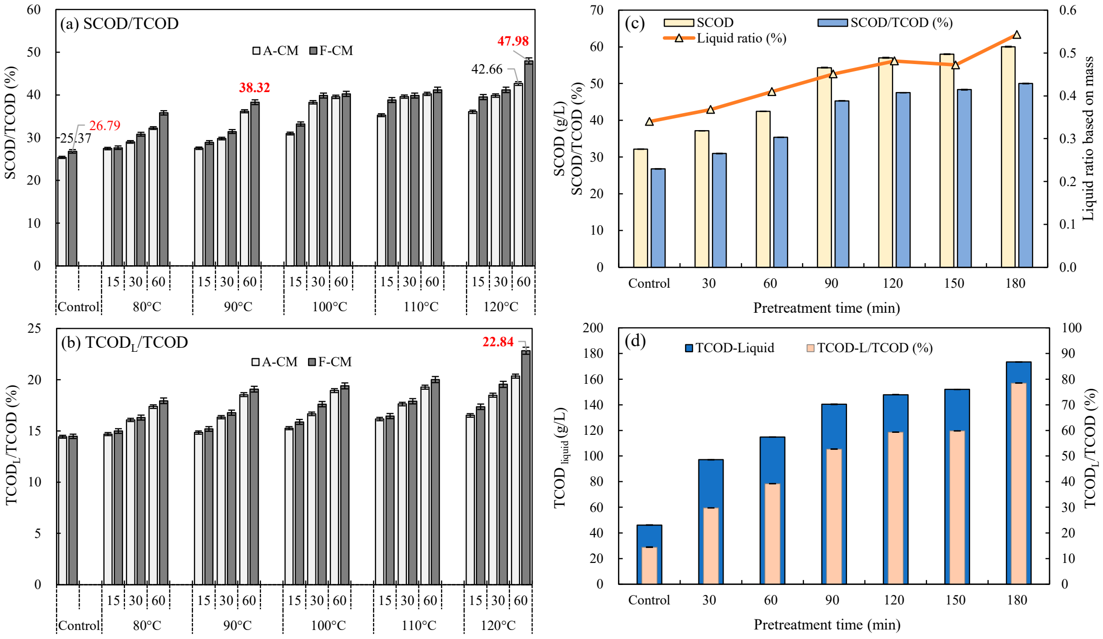


**Figure S1.** Hydrolysis efficiencies of chicken manure with thermal pretreatment under different temperature and time.

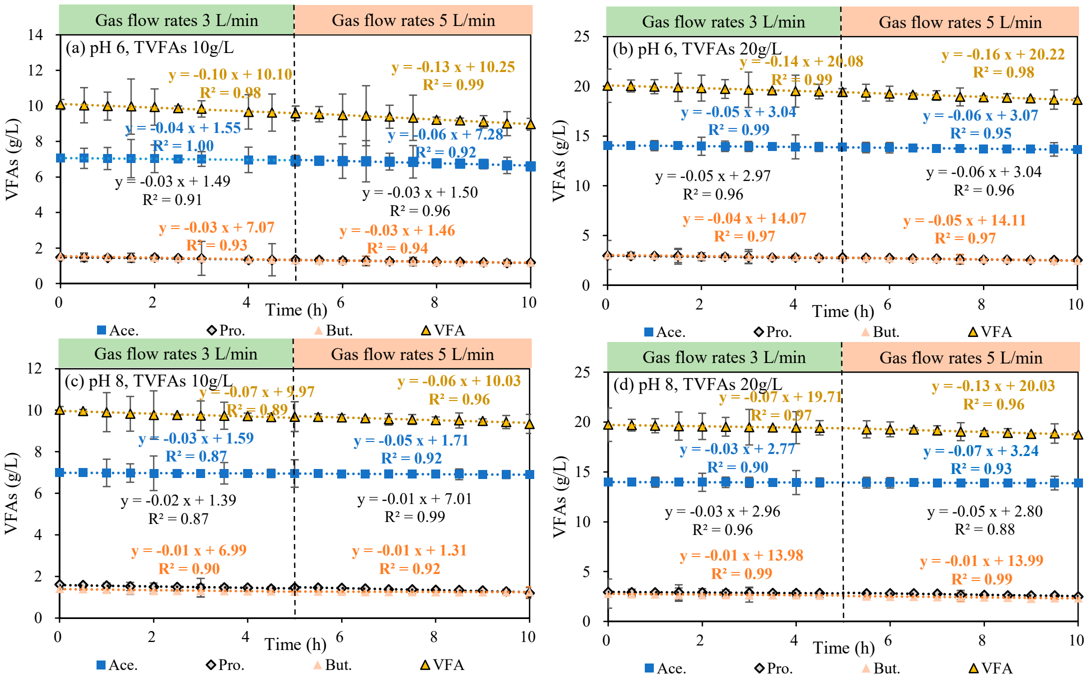


**Figure S2.** VFAs evaporation liner fit for different conditions of pH, $N_2$ flowing rates and initial VFAs concentration.

**Table S1.** VFAs evaporation test during different conditions of pH, $N_2$ flowing rates and initial VFAs concentration.

| | | pH 6.0 | | | | pH 8.0 | | | |
|---|---|---|---|---|---|---|---|---|---|
| Gas Flow Rates | | 3 L/min | | 5 L/min | | 3 L/min | | 5 L/min | |
| | | $k_1$ | $R^2$ | $k_2$ | $R^2$ | $k_1$ | $R^2$ | $k_2$ | $R^2$ |
| TVFA 10 g/L | Ace. | −0.04 | 0.93 | −0.06 | 0.92 | −0.03 | 0.90 | −0.05 | 0.99 |
| | Pro. | −0.03 | 0.91 | −0.03 | 0.96 | −0.02 | 0.87 | −0.01 | 0.92 |
| | but. | −0.03 | 1.00 | −0.03 | 0.94 | −0.01 | 0.87 | −0.01 | 0.92 |
| | VFA | −0.10 | 0.98 | −0.13 | 0.99 | −0.07 | 0.89 | −0.06 | 0.95 |
| TVFA 20 g/L | Ace. | −0.05 | 0.99 | −0.06 | 0.97 | −0.03 | 0.99 | −0.07 | 0.93 |
| | Pro. | −0.05 | 0.97 | −0.06 | 0.96 | −0.03 | 0.96 | −0.05 | 0.88 |
| | Ibu. | −0.04 | 0.96 | −0.05 | 0.95 | −0.01 | 0.90 | −0.01 | 0.99 |
| | VFA | −0.14 | 0.99 | −0.16 | 0.98 | −0.07 | 0.97 | −0.12 | 0.96 |

Notes: (1) The total volatile fatty acids (TVFA) consisted of acetate: propionate: butyrate = 7:1.5:1.5; (2) $k$ means the evaporated coefficient under different conditions, g/(L·h).
